# Cardiometabolic disease is associated with distinct proteomic signatures of acute disease activity in multiple sclerosis

**DOI:** 10.1016/j.neurot.2026.e01060

**Published:** 2026-09-10

**Authors:** Darin T. Okuda, Katy W. Burgess, Crystal M. Wright, Morgan C. Jones-McCreary, Isabella J. Huddleston, Jose R. Santoyo, Tom G. Punnen, Peter V. Sguigna, Lauren M. Tardo, Christine Lebrun-Frénay, Olaf Stüve, Diem H. Tran, Tatum M. Moog

**Affiliations:** a The University of Texas Southwestern Medical Center, Department of Neurology, Neuroinnovation Program, Multiple Sclerosis & Neuroimmunology Imaging Program, Dallas, TX, USA; b The University of Texas Southwestern Medical Center, Peter O'Donnell Jr. Brain Institute, Dallas, TX, USA; c The University of Texas Southwestern Medical Center School of Medicine, Dallas, TX, USA; d Université Nice Cote D’Azur, CRCSEP, Nice, France; e Dallas VA Medical Center, Department of Neurology, Dallas, TX, USA

**Keywords:** Multiple sclerosis, Cardiometabolic disease, Biomarkers, Disease-modifying therapy

## Abstract

Multiple sclerosis (MS) disease activity and treatment response may be influenced by systemic health factors, including cardiometabolic disease (CMD). This study evaluated how CMD influences inflammatory protein profiles and whether CMD modifies the effect of disease-modifying therapy (DMT). A retrospective study was conducted within a single academic MS center. All participants underwent commercial multi-analyte proteomic testing (Octave® Bioscience). CMD was defined as hypertension, type 2 diabetes mellitus, and/or dyslipidemia. Four groups were analyzed: untreated without CMD, untreated with CMD, DMT-treated without CMD, and DMT-treated with CMD. A multivariable variability index (MVI) was calculated across 18 age- and sex-adjusted proteins, and CMD burden (one, two, or three conditions) was assessed. A total of 287 MS individuals were included (84% White, 78% female). Among untreated individuals, those with CMD demonstrated significantly higher concentrations of proteins associated with acute MS disease activity, including MIP 3-alpha (CCL20) (p = 0.0078), CUB domain-containing protein 1 (CDCP1) (p = 0.0176), and TRAIL-R1 (TNFRSF10A) (p = 0.0002). In treated individuals, CMD was associated with higher levels of monokine induced by gamma interferon (CXCL9) (p = 0.0025), NfL (p = 0.0201), serpin family A member 9 (p = 0.0291), and TRAIL-R1 (p = 0.0002), as well as lower levels of protogenin (p = 0.0376). MVI values were elevated in both CMD groups. CMD was associated with higher odds of prior relapse and/or MRI activity within two years before proteomic testing (OR 5.48, 95% CI: [1.18–25.40], p = 0.0227). Protein concentrations increased with greater CMD burden. Clinically measurable proteins associated with acute MS disease activity are elevated in individuals with CMD and increase with comorbidity burden, in both DMT-treated and untreated groups.

## Introduction

Multiple sclerosis (MS) represents a spectrum of autoimmune demyelinating presentations that may be associated with permanent neurological disability [1], impacting nearly one million individuals within the U.S. [2]. The use of approved disease-modifying therapies (DMTs) represents a central strategy in suppressing acute clinical and radiological inflammatory demyelinating events and delaying progressive neurological decline. However, the magnitude of disease activity and treatment effect, both in untreated individuals and those receiving DMTs, may be influenced by broader systemic health factors, including cardiometabolic disease (CMD) such as hypertension [3], type 2 diabetes mellitus (T2DM) [4], and dyslipidemia [5]. Additionally, such conditions may increase risk for acute MS disease activity by altering the expression of proteins known to be associated with inflammatory activity and tissue damage [6].

Comorbid conditions may impact the timing and degree of disability at the diagnosis of MS [7], clinical relapses and MRI activity [8,9], treatment persistence [10], and disability progression [11]. The fundamental mechanisms that explain the observed associations may relate to the overproduction of pro-inflammatory cytokine and chemokines [12,13], resulting in metabolic reprogramming and altered immunometabolism within both peripheral and central nervous system immune compartments [14]. Additionally, individuals with MS appear to have a higher incidence of CMD when compared to unaffected adults [15]. However, the molecular pathways connecting CMD to acute MS disease activity remain poorly defined. Additionally, limited data exist on the impact of these conditions on MS-related inflammatory proteins and whether such systemic metabolic dysregulation may attenuate responsiveness to immunomodulatory or immunosuppressive treatments.

This study aims to investigate whether hypertension, T2DM, and/or dyslipidemia influence inflammatory and neuroprotective protein profiles that reflect immune activity and central nervous system (CNS) injury risk in MS, and to assess if these specific CMDs influence therapeutic response. We hypothesize that, within individuals with MS, i) CMDs are associated with increased prior disease activity, clinically and/or on MRI, ii) the presence of CMD will result in elevated inflammatory protein profiles, iii) protein profiles from individuals with CMD will differ according to DMT exposure, and iv) key protein biomarkers can be identified that may be useful for monitoring overall health in both treated and untreated individuals with MS who have coexisting cardiometabolic conditions. By clarifying the relationship between these chronic conditions and molecular markers of disease activity, this work may also provide evidence that optimizing systemic health could represent a modifiable pathway toward MS management beyond prescribed therapies.

## Methods

### Research participants

Individuals with relapsing-remitting MS evaluated in the Multiple Sclerosis and Neuroimmunology Clinic at The University of Texas Southwestern Medical Center (UTSWMC) were included in this study. All individuals with Octave® Multiple Sclerosis Disease Activity (MSDA) Test (Octave Bioscience, Menlo Park, CA) results from March 2025 to February 2026 were consecutively evaluated for study inclusion. All proteomic testing was performed as part of routine standard of care prior to the research study.

Inclusion criteria were i) individuals 18–65 years of age, ii) MS diagnosis by fellowship-trained neuroimmunologists, iii) available MSDA Test results, and iv) history of no change in DMT use over the past 6 months. Exclusion criteria were i) the presence of acute clinical relapses within 90 days of MSDA Test, ii) recent MRI activity related to MS within 90 days of MSDA Test, iii) compromised biomarker sample (i.e., shipping delays, compromised specimen during transport, expired stability window for the sample, etc.), iv) pregnant and postpartum women, and v) inadequate medical records (i.e., insufficient clinical documentation to reliably determine the presence or absence of CMD, unclear timing of DMT transition, or uncertain disease stability) for reliable assessment.

The study was approved by the Institutional Review Board at UTSWMC and a waiver of written consent was granted given the study design. The study was reported in accordance with the Strengthening the Reporting of Observational Studies in Epidemiology (STROBE) guidelines. A completed checklist is provided in Supplementary Table 1.

### Cardiometabolic disease definition

Cardiometabolic disease was defined in participants with MS by the presence of i) hypertension, ii) T2DM, and/or iii) dyslipidemia. Each participant was evaluated for the total number of CMDs present following the review of medical records from other specialists. A participant was considered to have these conditions based on the acquired clinical history provided by patients, if documented within recent medical records, and/or the use of on-label pharmacologic therapies specifically indicated for the management of these CMDs.

Four groups were formed and analyzed: i) untreated individuals with MS without history of CMD (Group 1), ii) untreated MS individuals with history of one or more CMDs (Group 2), iii) individuals with MS treated with DMTs without history of CMD (Group 3), and iv) MS individuals treated with DMTs with a history of CMD (Group 4).

### Acute disease activity definition

Acute disease activity encompassed both clinical relapses and MRI activity related to MS. Clinical relapses were defined as a new neurologic symptom consistent with CNS demyelination or worsening of a prior symptom, persisting for >24 h, that occurred in the absence of fever or acute illness. MRI activity related to MS was defined as the development of a new and/or newly enlarging T2-weighted hyperintense lesion and/or the presence of one or more gadolinium-enhancing lesions. Clinical relapses and MRI activity related to MS occurring within the two years prior to the date of MSDA testing were included.

### Statistical analysis

All analyses were performed in R (version 4.5.3) [16] and all figures were generated using the ggplot2 package. All 18 proteins studied were adjusted for sex and age using the methodology established for the Octave® MSDA Test [17].

Multivariable variability index (MVI) was computed as the square root of the sum of squared coefficients of variation of the 18 proteins studied. Mann-Whitney *U* test was used to compare outcomes across groups. Pairwise comparisons were performed in reference to the zero CMD group (i.e., no cardiometabolic conditions versus the presence of one CMD, no CMD versus the presence of two CMDs and no CMD versus three CMDs) in the treated and untreated groups using the Mann-Whitney *U* test and the resulting p-values were adjusted using the Benjamini and Hochberg correction to account for multiple comparisons within each protein and independently within the treated and untreated groups. Pairwise comparisons were also performed between those with one, two, and three CMDs, irrespective of DMT treatment status, and resulting p-values were adjusted using the Benjamini and Hochberg correction to account for multiple comparisons within each protein.

To determine if the odds of a clinical relapse and/or MRI activity in the two years prior to Octave testing differed across the groups studied, a logistic regression model was fitted to this outcome, controlling for age, sex at birth, and disease duration. Tukey's multiple comparisons were performed to test for pairwise differences between groups and p-values were adjusted using the single-step method to account for multiple comparisons.

Significance was defined as p < 0.05. Adjustments for multiple comparisons were applied only to the prespecified comparisons described above; all other analyses were unadjusted.

## Results

**Fig. 1:**
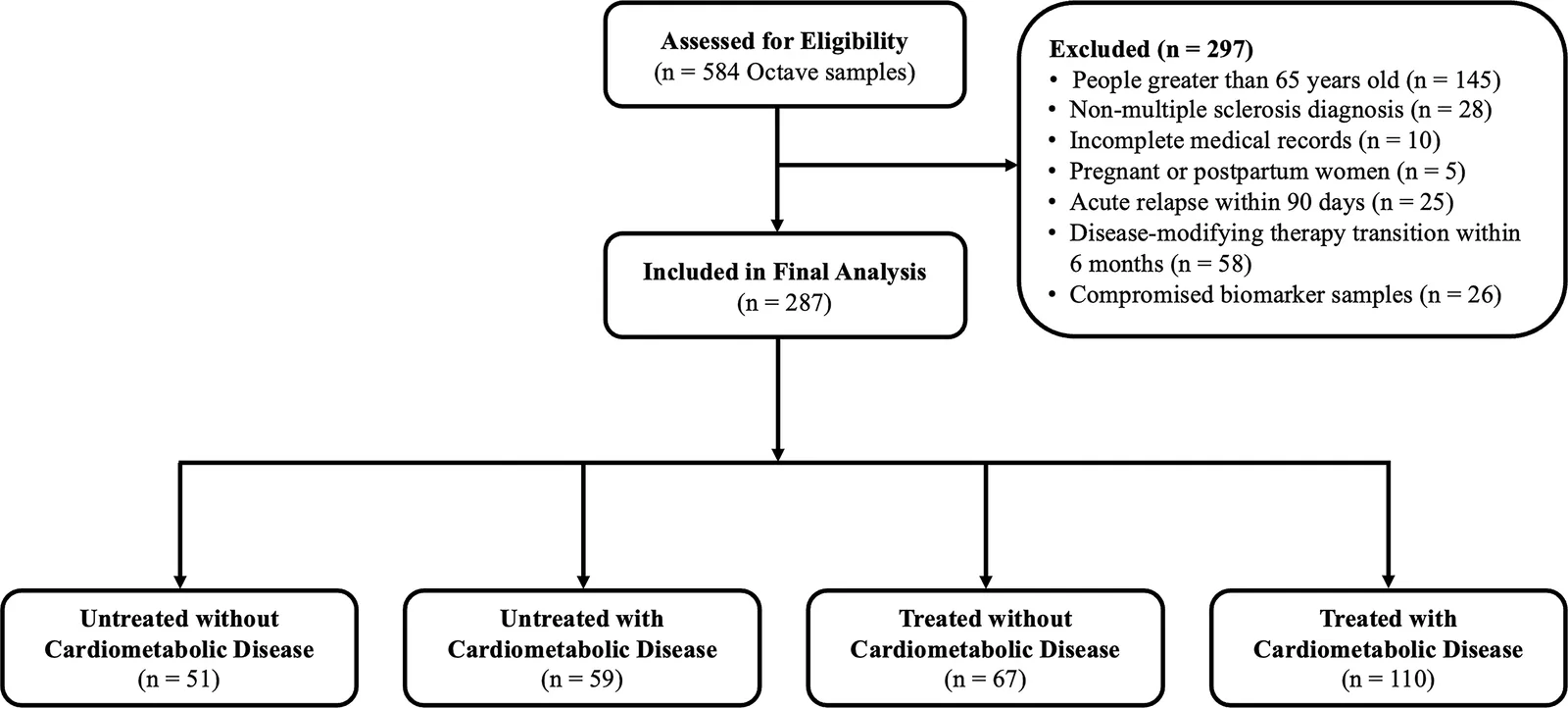
Flow diagram summarizing participant assessment for eligibility, exclusions with reasons, and allocation within the four study groups analyzed.

A total of 287 individuals fulfilling the 2017 McDonald criteria [18] for MS were studied and stratified by the presence of CMD and treatment status (Fig. 1). The majority of participants were White (84%) and female (78%) with a median age of 48.5 years (range: 19.7–65.0). Within the cohort, 169 individuals (58.9%) had at least one CMD. Dyslipidemia (n = 127) was most prevalent, followed by hypertension (n = 123) and T2DM (n = 31). Among the untreated cohorts, 51 individuals without CMD had a median disease duration of 8.0 years (0.5–33.2 years), while 59 individuals with CMD (44 with hypertension, 41 with dyslipidemia, and 15 with T2DM) had a median duration of 12.5 years (0.4–34.0 years). Within the treated cohorts, 67 individuals without CMD had a median disease duration of 8.5 years (0.4–32.0 years), whereas 110 individuals with CMD – 86 with dyslipidemia, 79 with hypertension, and 16 with T2DM - had a median duration of 10.4 years (0.4–33.0 years). Table 1 provides a summary of demographic and clinical information by group.

**Table 1:** Demographic and clinical information by group.

	Untreated Multiple Sclerosis without Cardiometabolic Disease	Untreated Multiple Sclerosis with Cardiometabolic Disease	Treated Multiple Sclerosis without Cardiometabolic Disease	Treated Multiple Sclerosis with Cardiometabolic Disease
Number of subjects (n)	51	59	67	110
Number of subjects with 1 CMD^a^ (%)	0 (0.0)	28 (47.5)	0 (0.0)	52 (47.3)
Number of subjects with 2 CMDs^a^ (%)	0 (0.0)	21 (35.6)	0 (0.0)	46 (41.8)
Number of subjects with 3 CMDs^a^ (%)	0 (0.0)	10 (16.9)	0 (0.0)	12 (10.9)
Median (range) age (years)	46.9 (25.3, 65.0)	56.0 (25.6, 65.0)	41.7 (19.7, 62.8)	54.3 (25.5, 65.0)
Females, n (%)	43 (84.3)	43 (72.9)	52 (77.6)	87 (79.1)
Ethnicity, n (%)				
Hispanic	1 (1.9)	9 (15.3)	5 (7.5)	8 (7.3)
Non-Hispanic	50 (98.1)	50 (84.7)	62 (92.5)	102 (92.7)
Race, n (%)				
White	49 (96.1)	47 (79.7)	55 (82.0)	89 (80.9)
African American	1 (1.9)	8 (13.6)	6 (9.0)	16 (14.5)
Asian	1 (1.9)	3 (5.1)	5 (7.5)	4 (3.6)
Native American	0 (0.0)	1 (1.7)	1 (1.5)	1 (0.9)
Median (range) disease duration (years)	8.0 (0.5, 33.2)	12.5 (0.4, 34.0)	8.5 (0.4, 32.0)	10.4 (0.4, 33.0)
Median (range) treatment duration (years)			2.7 (0.4, 15.3)	3.2 (0.4, 14.6)
Disease modifying therapy types, n (%)				
cladribine			0 (0.0)	4 (3.6)
dimethyl fumarate			4 (6.0)	9 (8.2)
diroximel fumarate			3 (4.5)	6 (5.5)
fingolimod			6 (9.0)	16 (14.5)
glatiramer acetate			10 (14.9)	10 (9.1)
interferon beta-1 alpha thrice weekly			1 (1.5)	1 (0.9)
interferon beta-1 alpha weekly			0 (0.0)	2 (1.8)
interferon beta-1 beta			0 (0.0)	1 (0.9)
natalizumab			2 (3.0)	5 (4.5)
ocrelizumab			22 (32.8)	27 (24.5)
ofatumumab			11 (16.4)	7 (6.4)
ozanimod			1 (1.5)	8 (7.3)
ponesimod			1 (1.5)	0 (0.0)
rituximab			0 (0.0)	2 (1.8)
siponimod			1 (1.5)	0 (0.0)
teriflunomide			4 (6.0)	9 (8.2)
ublituximab-xiiy			1 (1.5)	3 (2.7)

CMD^a^ = cardiometabolic disease.

When comparing untreated individuals, those with CMD had significantly higher odds of experiencing a preceding acute clinical relapse and/or MRI activity related to MS within the two years prior to their first proteomic study than those without (odds ratio (OR): 5.48, 95% confidence interval (CI): [1.18, 25.40], p = 0.0227). In contrast, among individuals being treated with DMTs, no significant difference was observed in the odds of acute disease activity between those with and without CMD (OR: 1.30, 95% CI: [0.20, 8.68], p = 0.9841).

In untreated individuals with MS, no significant difference in the composite disease activity score (DAS) was observed between those with or without CMD (p = 0.8073). Significantly higher concentrations of MIP 3-Alpha (CCL20) (p = 0.0078), a protein directly correlated with acute disease activity, was observed in individuals with CMD when compared to those without. However, significantly higher concentrations of tumor necrosis factor receptor superfamily member 10 A (TNFRSF10A) (p = 0.0002) and CUB domain-containing protein 1 (CDCP1) (p = 0.0176), proteins inversely correlated with acute disease activity, were observed in individuals with CMD compared to those without CMD (Table 2). A higher MVI, reflecting greater variability among the 18 proteins, was also observed in those with CMD (4.23) when compared with individuals without CMD (2.85).

**Table 2:** Octave Multiple Sclerosis Disease Activity Test protein concentrations (median (range)) in untreated individuals with multiple sclerosis, with and without cardiometabolic disease.

	Untreated Multiple Sclerosis without Cardiometabolic Disease	Untreated Multiple Sclerosis with Cardiometabolic Disease	p-value	Rank Biserial Correlation (95% CI)
**Proteins Associated with Acute Disease Activity**				
Cluster of Differentiation 6 (CD6) pg/mL	108.84 (32.05, 207.33)	104.99 (47.90, 367.39)	0.9594	−0.01 (−0.22, 0.21)
Contactin 2 (CNTN2) ng/mL	−1.49 (−2.47, 0.90)	−1.32 (−2.34, 3.21)	0.9761	−0.00 (−0.22, 0.21)
C-X-C motif chemokine ligand 13 (CXCL13) pg/mL	41.93 (22.63, 182.87)	42.98 (7.62, 357.48)	0.7828	−0.03 (−0.24, 0.18)
Glial fibrillary acidic protein (GFAP) pg/mL	174.63 (53.83, 612.72)	170.12 (65.78, 596.98)	0.8809	−0.02 (−0.23, 0.20)
Leucine-rich repeat transmembrane (FLRT2) pg/mL	107.64 (65.96, 194.92)	109.74 (58.83, 225.16)	0.8596	−0.02 (−0.23, 0.19)
MIP 3-Alpha (CCL20) pg/mL	5.76 (1.82, 98.97)	8.63 (2.45, 93.99)	0.0078	−0.30 (−0.48, −0.09)
Monokine induced by gamma interferon (CXCL9) pg/mL	38.71 (16.47, 188.68)	47.69 (17.60, 129.82)	0.1486	−0.16 (−0.36, 0.06)
Myelin oligodendrocyte glycoprotein (MOG) pg/mL	27.09 (10.65, 64.28)	28.48 (5.91, 59.23)	0.9427	−0.01 (−0.22, 0.21)
Neurofilament light (NfL) pg/mL	6.33 (2.54, 21.12)	7.57 (2.46, 111.49)	0.0962	−0.18 (−0.38, 0.03)
Osteopontin (OPN) ng/mL	17.02 (3.09, 44.16)	17.52 (4.70, 46.19)	0.4555	−0.08 (−0.29, 0.13)
Protogenin (PRTG) pg/mL	118.04 (75.18, 500.01)	113.22 (60.72, 187.84)	0.5271	0.07 (−0.15, 0.28)
Serpin family A member 9 (SERPINA9) pg/mL	47.20 (3.56, 278.72)	52.04 (16.69, 235.10)	0.8903	−0.02 (−0.23, 0.20)
**Proteins inversely Associated with Acute Disease Activity**				
Amyloid beta precursor like protein 1 (APLP1) ng/mL	9.84 (1.79, 20.41)	9.24 (1.22, 31.20)	0.2260	0.13 (−0.08, 0.34)
B-cell Activating factor (TNFSF13B) ng/mL	1.36 (−0.64, 8.03)	0.86 (−1.47, 41.09)	0.1379	0.16 (−0.05, 0.37)
CUB domain-containing protein 1 (CDCP1) pg/mL	67.65 (24.15, 485.15)	85.61 (39.37, 325.19)	0.0176	−0.26 (−0.45, −0.05)
Interleukin 12 B (IL-12 B) pg/mL	93.54 (35.25, 370.91)	105.64 (12.55, 3042.27)	0.1893	−0.15 (−0.35, 0.07)
Osteoprotegerin (OPG) ng/mL	−2.08 (−2.35, −0.95)	−2.03 (−2.40, −0.52)	0.2860	−0.12 (−0.32, 0.10)
TRAIL-R1 (TNFRSF10A) pg/mL	3.91 (1.78, 7.24)	5.06 (2.16, 9.88)	0.0002	−0.42 (−0.58, −0.22)

Similarly, among treated individuals with MS with or without CMD, no significant difference was observed in the DAS. Significantly higher concentrations of proteins correlated with acute disease activity were seen in monokine induced by gamma interferon (CXCL9) (p = 0.0025), neurofilament light (NfL) (p = 0.0201), serpin family A member 9 (SERPINA9) (p = 0.0291), whereas protogenin (PRTG) (p = 0.0376) was found to be significantly lower among those with CMD compared to those without. In contrast, significantly higher levels of TNFRSF10A (p = 0.0002) were observed among those with CMD compared to those without CMD (Table 3). A higher MVI value for all proteins was also observed in treated individuals with CMD (4.10) when compared to those without (2.87).

**Table 3:** Octave Multiple Sclerosis Disease Activity Test protein concentrations (median (range)) in treated individuals with multiple sclerosis, with and without cardiometabolic disease.

	Treated Multiple Sclerosis without Cardiometabolic Disease	Treated Multiple Sclerosis with Cardiometabolic Disease	p-value	Rank Biserial Correlation (95% CI)
**Proteins Associated with Acute Disease Activity**				
Cluster of Differentiation 6 (CD6) pg/mL	92.93 (26.94, 344.00)	87.79 (39.96, 318.19)	0.8643	0.02 (−0.16, 0.19)
Contactin 2 (CNTN2) ng/mL	−1.59 (−2.36, 1.99)	−1.60 (−2.75, 0.74)	0.7031	0.03 (−0.14, 0.21)
C-X-C motif chemokine ligand 13 (CXCL13) pg/mL	39.02 (14.27, 336.00)	35.01 (1.61, 489.88)	0.0666	0.16 (−0.01, 0.33)
Glial fibrillary acidic protein (GFAP) pg/mL	147.41 (71.19, 379.38)	169.93 (55.40, 506.38)	0.0593	−0.17 (−0.33, 0.00)
Leucine-Rich repeat transmembrane (FLRT2) pg/mL	113.75 (43.26, 202.62)	108.41 (61.53, 239.65)	0.2989	0.09 (−0.08, 0.26)
MIP 3-Alpha (CCL20) pg/mL	5.58 (−0.03, 47.57)	5.96 (1.15, 382.40)	0.2334	−0.11 (−0.28, 0.07)
Monokine induced by gamma interferon (CXCL9) pg/mL	33.78 (10.80, 530.69)	43.83 (13.54, 1083.25)	0.0025	−0.27 (−0.43, −0.10)
Myelin oligodendrocyte glycoprotein (MOG) pg/mL	26.69 (7.89, 61.60)	26.45 (10.56, 56.82)	0.5185	−0.06 (−0.23, 0.12)
Neurofilament light (NfL) pg/mL	5.14 (2.48, 14.19)	5.68 (2.40, 21.35)	0.0201	−0.21 (−0.37, −0.04)
Osteopontin (OPN) ng/mL	14.28 (5.74, 33.37)	15.33 (5.54, 69.18)	0.1517	−0.13 (−0.30, 0.05)
Protogenin (PRTG) pg/mL	124.07 (79.75, 222.48)	117.93 (68.60, 264.18)	0.0376	0.19 (0.01, 0.35)
Serpin family A member 9 (SERPINA9) pg/mL	26.42 (5.71, 184.37)	39.13 (5.29, 511.71)	0.0291	−0.20 (−0.36, −0.02)
**Proteins Inversely Associated with Acute Disease Activity**				
Amyloid beta precursor like protein 1 (APLP1) ng/mL	9.69 (1.65, 51.37)	8.64 (0.20, 28.87)	0.2690	0.10 (−0.08, 0.27)
B-cell Activating factor (TNFSF13B) ng/mL	2.84 (−0.45, 14.41)	2.16 (−1.24, 24.35)	0.5768	0.05 (−0.12, 0.22)
CUB domain-containing protein 1 (CDCP1) pg/mL	68.75 (31.56, 251.79)	79.78 (26.32, 402.57)	0.0675	−0.16 (−0.33, 0.01)
Interleukin 12 B (IL-12 B) pg/mL	90.86 (8.07, 350.96)	101.40 (16.89, 296.35)	0.1655	−0.12 (−0.29, 0.05)
Osteoprotegerin (OPG) ng/mL	−2.10 (−2.46, −1.28)	−2.01 (−2.44, −0.30)	0.2111	−0.11 (−0.28, 0.06)
TRAIL-R1 (TNFRSF10A) pg/mL	3.87 (2.06, 8.14)	4.76 (1.96, 14.36)	0.0002	−0.34 (−0.49, −0.18)

**Fig. 2:**
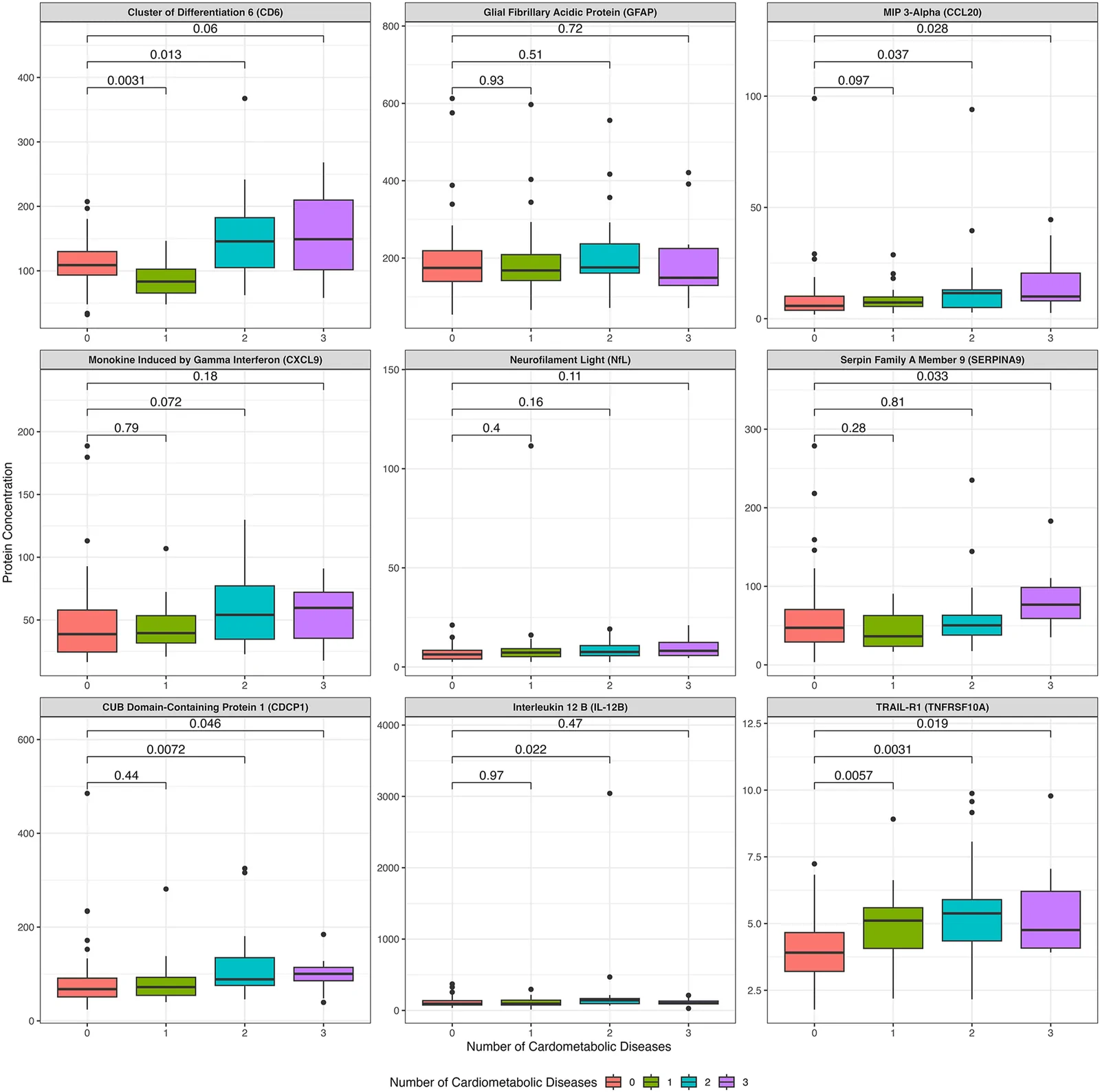
Boxplots showing the concentrations of nine individual proteins of interest in all untreated multiple sclerosis individuals. Individuals with no cardiometabolic disease (CMD) were compared to those with one, two, and three diseases to evaluate the impact of comorbidity burden on protein concentrations. P-values reflect pairwise comparisons adjusted for multiple testing.

**Fig. 3:**
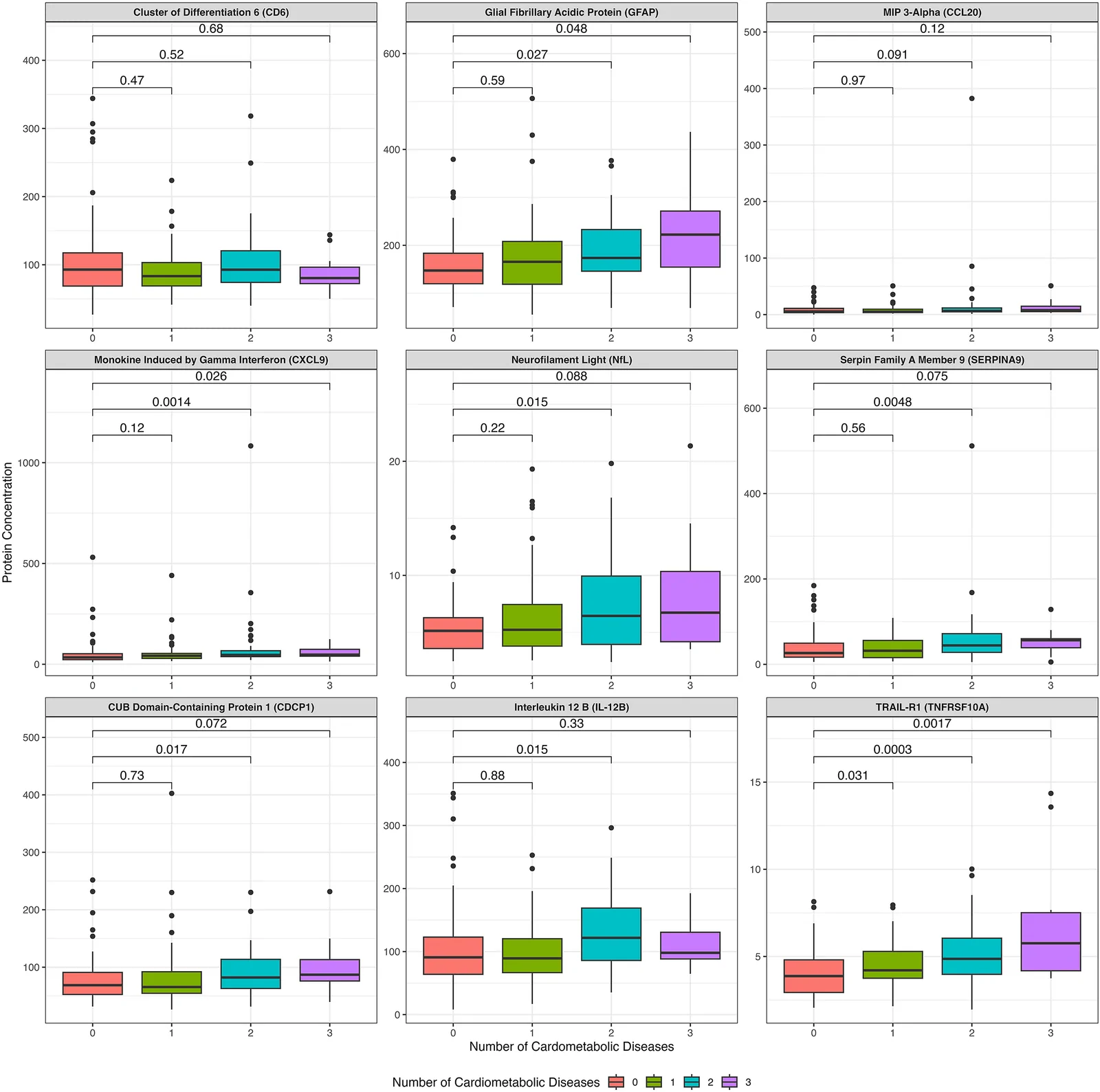
Boxplots illustrating the concentrations of nine distinct proteins of interest in multiple sclerosis individuals treated with disease-modifying therapies. Individuals with no cardiometabolic disease (CMD) were compared to those with one, two, and three diseases to assess the cumulative impact of comorbidity burden on protein levels. P-values reflect pairwise comparisons adjusted for multiple testing.

In the comparison of untreated MS individuals with and without CMD, the number of CMDs influenced both proteins correlated and inversely correlated with acute MS disease activity (Fig. 2). Among individuals not receiving DMT, several proteins that were not elevated in the presence of a single CMD revealed higher concentrations in individuals with two or three CMDs, including CCL20, CDCP1, and SERPINA9. Consistent with these findings, all treated individuals with more than one CMD exhibited consistently higher protein concentrations with increasing CMD burden for GFAP, CXCL9, and TNFRSF10A. (Fig. 3, Table 2 and Table 3). Supplementary figures provide acquired data for each protein.

## Discussion

In this study, CMD was associated with select differences in protein concentrations linked to inflammatory disease activity in both treated and untreated individuals with MS. Untreated individuals with CMD had higher odds of experiencing a clinical relapse and/or MRI activity during the two years preceding the first proteomic test. Elevated protein concentrations were observed in studied individuals with two or three CMDs when compared to a single CMD, suggesting a cumulative effect with comorbidity burden. Additionally, inflammatory protein concentrations remained higher in individuals with CMD despite treatment. Together, these findings suggest that CMD is associated with clinically meaningful differences in MS, and that attention to general health is important in the comprehensive management of individuals with MS.

The commercially available multi-analyte panel used in this study, validated both analytically [17] and clinically [6], enabled the assessment of 18 proteins, 12 of which are correlated with acute MS disease activity. The DAS, calculated using a proprietary algorithm, ranges from 1.0 to 10.0 in 0.5 increments. Higher scores indicate greater acute disease activity risk with scores ranging from 4.5 to 10.0 associated with a 4.49-fold increase in the odds of having >1 gadolinium-enhancing lesion on MRI, compared to lower range scores of 1.0–4.0, while scores of 7.5–10.0 correspond to a 20.99-fold increase in the odds of having >2 gadolinium-enhancing lesions when compared to the lower range of 1.0–7.0 [6]. In addition to the DAS, the concentrations of all 18 proteins are provided.

**Fig. 4:**
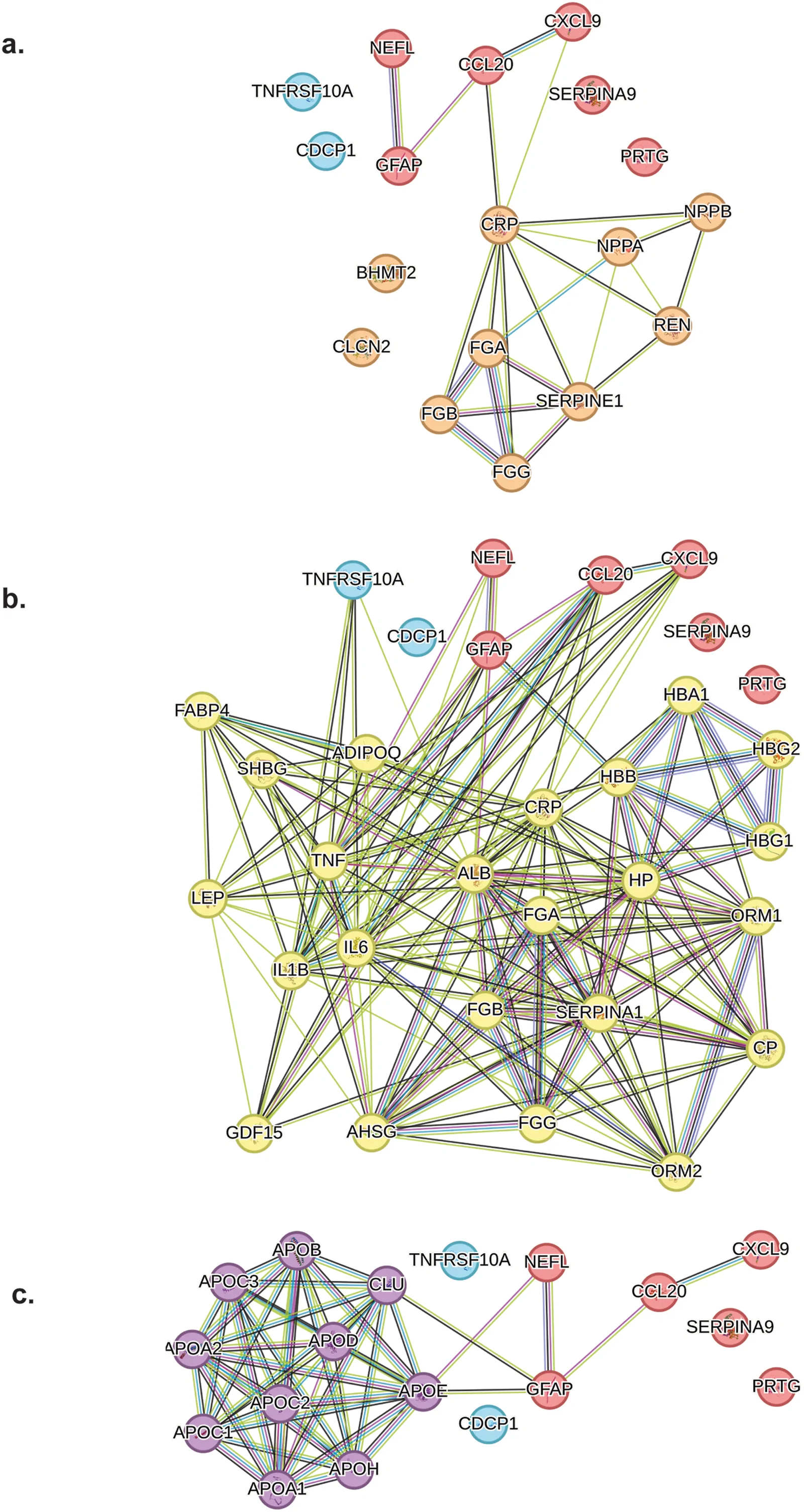
STRING network analysis illustrating known and predicted protein–protein interactions between the eight significant proteins identified in this study - CUB domain–containing protein 1 (CDCP1), MIP 3-Alpha (CCL20), monokine induced by gamma interferon (CXCL9), glial fibrillary acidic protein (GFAP), neurofilament light chain (NEFL), protogenin (PRTG), serpin family A member 9 (SERPINA9), and tumor necrosis factor receptor superfamily member 10 A (TNFRSF10A) (proteins correlated with acute disease activity in red and inversely correlated in blue) - and proteins associated with **(A)** hypertension (chloride voltage-gated channel 2 (CLCN2), atrial natriuretic peptide (NPPA), B-type natriuretic peptide B (NPPB), C-reactive protein (CRP), fibrinogen alpha chain (FGA), fibrinogen beta chain (FGB), fibrinogen gamma chain (FGG), betaine--homocysteine S-methyltransferase 2 (BHMT2), serpin peptidase inhibitor, clade E (SERPINE1), and renin (REN)), **(B)** type 2 diabetes mellitus (adiponectin (ADIPOQ), Alpha-2-HS-glycoprotein chain A (AHSG), serum albumin (ALB), ceruloplasmin (CP), C-reactive protein (CRP), fatty acid–binding protein 4 (FABP4), fibrinogen alpha chain (FGA), fibrinogen beta chain (FGB), fibrinogen gamma chain (FGG), growth differentiation factor 15 (GDF15), hemoglobin subunit alpha 1 (HBA1), hemoglobin subunit beta (HBB), hemoglobin subunit gamma 1 (HBG1), hemoglobin subunit gamma 2 (HBG2), haptoglobin alpha chain (HP), IL-1β (interleukin-1 beta), IL-6 (interleukin-6), LEP (leptin), orosomucoid 1(ORM1), orosomucoid 2 (ORM2), alpha-1 antitrypsin (SERPINA1), sex hormone–binding globulin (SHBG), and tumor necrosis factor alpha (TNF-α)), and **(C)** dyslipidemia (apolipoprotein A1 (ApoA1), apolipoprotein A2 (ApoA2), apolipoprotein B (ApoB), apolipoprotein C1 (ApoC1), apolipoprotein C2 (ApoC2), apolipoprotein C3 (ApoC3), apolipoprotein D (ApoD), apolipoprotein E (ApoE), apolipoprotein H (ApoH), and clusterin alpha chain (CLU)). Edges represent protein–protein associations derived from multiple evidence sources: sky-blue lines indicate interactions curated from databases; purple lines represent experimentally determined interactions; green lines denote predicted interactions based on gene neighborhood; red lines indicate predicted interactions based on gene fusion; blue lines indicate predicted interactions based on gene co-occurrence; lime-green lines correspond to evidence from text mining; black lines indicate co-expression; and violet-blue lines represent protein homology.

Several biological mechanisms support the relationship between CMD and MS disease worsening. Astrocytes regulate blood-brain barrier integrity via perivascular end feet, stabilizing tight junctions and controlling water flux [19]. Cardiometabolic stress may activate immune cells resulting in an increase in systemic inflammation, compromising blood-brain barrier integrity [[20], [21], [22], [23], [24], [25], [26]]. Supportive of this was the observation of increased contrast enhancement on MRI which has been observed in individuals with well-controlled T2DM [27], and less blood-brain barrier leakage in well-controlled individuals with hypertension [28]. CMD therefore may be expected to modulate levels of proteins related to various inflammatory mechanisms that may also have relevance in biomarker assays measuring acute disease activity in MS. The differential protein signatures observed between treated and untreated individuals suggest that CMD interacts with MS biology in a treatment-dependent manner. Proteins associated with hypertension, T2DM, and dyslipidemia support a mechanistic link between these chronic conditions and MS-related disease activity (Fig. 4). Untreated individuals with CMD exhibited higher levels of neuroinflammatory chemokines (CCL20, CXCL9), suggesting greater inflammatory activity in the absence of DMT exposure. Conversely, elevated concentrations of CXCL9, NfL, and SERPINA9, along with lower PRTG concentrations were observed among treated individuals with CMD, suggesting that these conditions contribute to proteins linked with immunomodulation and neuroaxonal integrity despite treatment. This was supported by the elevation of NfL seen in treated individuals with CMD that were not seen in treated individuals without CMD. These findings may be consistent with an association between CMD and neuroaxonal injury proteins, potentially mediated through pathways that are not fully attenuated by anti-inflammatory DMT treatment.

In both treated and untreated individuals with CMD, TNFRSF10A, a cell-surface receptor that binds to the cytotoxic TRAIL ligand, was persistently elevated [29]. While the biological role of TNFRSF10A in MS is not fully established, proposed mechanisms include modulation of auto-reactive lymphocytes [30] and increased oligodendrocyte vulnerability [31]. However, the observed elevation of TNFRSF10A despite DMT exposure suggests that expression may be influenced by CMD, when present, rather than biological mechanisms associated with MS alone. Given that TRAIL signaling has been noted in several cardiometabolic conditions, including diabetes [32], hypercholesterolemia [33], and obesity-related heart failure [34], the observed elevation in TNFRSF10A across treatment strata may reflect compensatory receptor upregulation at the cell surface or receptor membrane shedding in response to cellular stress induced by cardiometabolic disease [35].

Our findings suggest a complex intersection between cardiometabolic stress and immune modulation, providing a reason for the lack of significant difference observed in the DAS between the groups studied. Although several biomarkers in the MSDA Test have biologically plausible connections to CMD, expression may be dependent on temporal and pathophysiologic windows. Chemokines CCL20 and CXCL9 may be more responsive to acute or fluctuating immune activation [36,37], whereas NfL reflects neuroaxonal injury that is influenced by age, disease stage, other neurologic conditions, and treatment exposure [38]. As the proteins included in the MSDA Test were selected to reflect biological processes associated with MS disease activity, the absence of widespread differences across the proteomic profile suggests that protein-level associations observed with CMD are limited to select pathways that overlap with biological processes involved in MS.

Within routine clinical practice, a subset of individuals with MS prefer not to receive DMT treatment. This may be the result of a preference toward more natural approaches to care or a reflection of the paradox of choice in which an abundance of treatments results in intrinsically lower ability to decide on a specific selection and increased anxiety [39]. For others, treatment may not be a viable option due to existing medical conditions for which immunotherapy is contraindicated, healthcare coverage, financial constraints, or cultural beliefs that conflict with prescribed options. The findings here suggest that measurable changes in acute inflammatory proteins may be detected even in the absence of treatment, emphasizing the potential utility of these biomarkers for both MS management and possibly broader chronic disease monitoring.

**Fig. 5:**
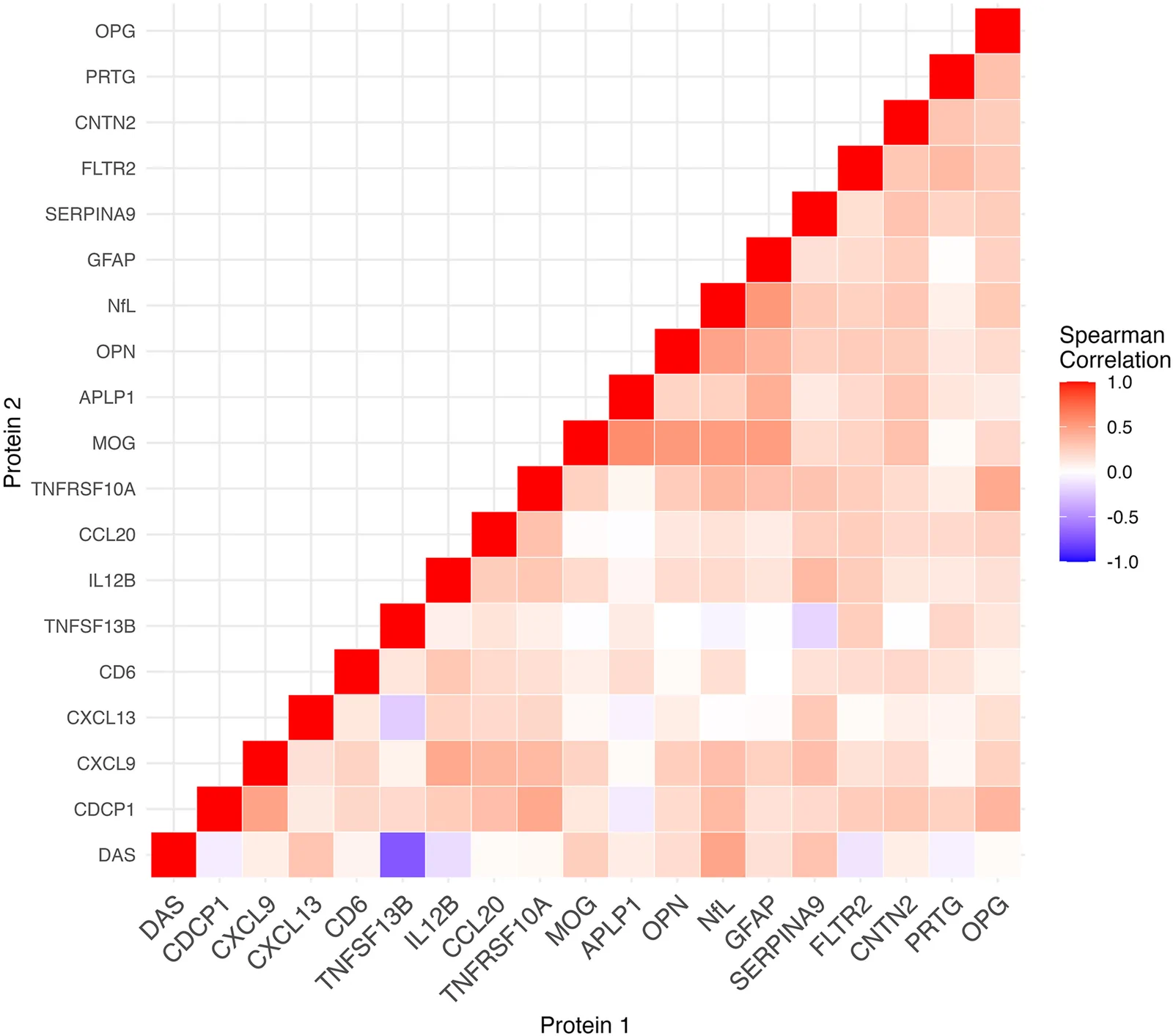
Spearman rank correlation matrix of the 18 proteins included in the Multiple Sclerosis Disease Activity Test. Correlation coefficients are displayed as a triangular heatmap, with each cell representing the pairwise Spearman correlation between two proteins. Color intensity reflects the strength and direction of the correlation, ranging from −1 (blue, strong negative correlation) to +1 (red, strong positive correlation), with white indicating little or no correlation. Only the lower triangular portion of the correlation matrix is shown to avoid redundant pairwise comparisons.

The presented findings should be interpreted in the context of limitations. First, the study cohort was obtained from a specialty center within an academic institution and included subjects may not be generalizable to other practices. Second, 84% of study participants were White and the inclusion of a more racially diverse group may yield different outcomes given the known differences in CMD prevalence [40]. Third, measures of social determinants of health were not included and may represent unmeasured confounding. Fourth, the level of control and duration of cardiometabolic conditions were not measured and the influence of these factors may have impacted our results. Fifth, retrospective cross-sectional data were analyzed in this work. The inclusion of additional time points would allow for a more detailed characterization of the dynamic rates of change of the proteins highlighted relative to CMD activity. Lastly, correlations among the proteins included in the MVI determination may result in overlapping contributions to the index. Accordingly, the MVI is reported as a descriptive measure and should be interpreted in the context of the correlation structure among the proteins (Fig. 5).

The exact contribution of improvements in general health on MS outcomes remains incompletely defined. Our reported findings raise the possibility that lifestyle interventions targeting metabolic health may favorably modulate known proteins associated with acute MS disease activity, potentially improving long-term outcomes by reducing relapse risk, addressing factors that may impact treatment responses, and ultimately reducing healthcare costs. The potential impact on neurodegeneration adds further intrigue [[41], [42], [43]]. If indeed true, people with MS on treatment or those unable to receive treatment may directly modify their own drivers of inflammatory risk by improvements in general health. An expanded integrated approach to care that involves cardiometabolic management may also represent an advancement over current models, aligning with successful multidisciplinary approaches employed in other specialties in neurology and serving as an adjunctive therapeutic strategy.

## Author contributions

D.T.O. conceptualized the study and contributed to methodology, validation, and drafting of the manuscript. K.W.B., C.M.W., I.J.H., J.R.S., T.G.P., P.V.S., L.M.T., C.L.-F., D.H.T., and T.M.M., curated data, participated in investigation, and contributed to reviewing and editing the manuscript. M.C.M. curated and formally analyzed the data and contributed to drafting as well as reviewing and editing the manuscript. O.S. contributed to data curation, methodology, and manuscript review. All authors read and approved the final manuscript.

## Data availability

The datasets generated and analyzed during the current study are available from the corresponding author, D.T.O., on reasonable request and following successful completion of a data transfer agreement with UTSWMC.

## Funding

The author(s) received no financial support for the research, authorship, and/or publication of this article.

## Declaration of competing interest

The authors declare the following financial interests/personal relationships which may be considered as potential competing interests:

Darin T. Okuda reports a relationship with Amgen that includes: consulting or advisory. Darin T. Okuda reports a relationship with Biogen that includes: consulting or advisory. Darin T. Okuda reports a relationship with Cortechs. ai that includes: consulting or advisory. Darin T. Okuda reports a relationship with EMD Serono that includes: consulting or advisory. Darin T. Okuda reports a relationship with Genentech that includes: consulting or advisory. Darin T. Okuda reports a relationship with Genzyme Corporation that includes: consulting or advisory. Darin T. Okuda reports a relationship with Immunic Therapeutics that includes: consulting or advisory. Darin T. Okuda reports a relationship with Moderna that includes: consulting or advisory. Darin T. Okuda reports a relationship with Octave Bioscience Inc that includes: consulting or advisory. Darin T. Okuda reports a relationship with Pfizer that includes: consulting or advisory. Darin T. Okuda reports a relationship with Zenas BioPharma that includes: consulting or advisory. Darin T. Okuda reports a relationship with Alexion that includes: funding grants. Darin T. Okuda reports a relationship with Novartis that includes: funding grants. Katy W. Burgess reports a relationship with EMD Serono that includes: consulting or advisory. Katy W. Burgess reports a relationship with Sanofi that includes: consulting or advisory. Crystal M. Wright reports a relationship with EMD Serono that includes: consulting or advisory. Crystal M. Wright reports a relationship with Genentech that includes: consulting or advisory. Crystal M. Wright reports a relationship with TG Therapeutics that includes: consulting or advisory. Peter V. Sguigna reports a relationship with Amgen that includes: consulting or advisory. Peter V. Sguigna reports a relationship with Bristol-Myers Squibb that includes: consulting or advisory. Peter V. Sguigna reports a relationship with EMD Serono that includes: consulting or advisory. Peter V. Sguigna reports a relationship with Genentech that includes: consulting or advisory. Peter V. Sguigna reports a relationship with Horizon Therapeutics that includes: consulting or advisory. Peter V. Sguigna reports a relationship with Clene Nanomedicine that includes: funding grants. Peter V. Sguigna reports a relationship with Genentech that includes: funding grants. Peter V. Sguigna reports a relationship with Novartis that includes: funding grants. Peter V. Sguigna reports a relationship with Zenas BioPharma that includes: funding grants. Lauren M. Tardo reports a relationship with EMD Serono that includes: consulting or advisory. Lauren M. Tardo reports a relationship with CanDoMS that includes: consulting or advisory. Lauren M. Tardo reports a relationship with MJH Life Sciences that includes: consulting or advisory. Lauren M. Tardo reports a relationship with NeurologyLive that includes: consulting or advisory. Lauren M. Tardo reports a relationship with The MOG Project that includes: non-financial support. Olaf Stuve reports a relationship with Cellarity that includes: consulting or advisory. Olaf Stuve reports a relationship with Lilly that includes: consulting or advisory. Olaf Stuve reports a relationship with Octave Bioscience that includes: consulting or advisory. Olaf Stuve reports a relationship with Zenas BioPharma that includes: consulting or advisory. Olaf Stuve reports a relationship with Therapeutic Advances in Neurological Disorders that includes: board membership. Olaf Stuve reports a relationship with Expert Review of Clinical Immunology that includes: board membership. Olaf Stuve reports a relationship with Current Treatment Options in Neurology that includes: board membership. Olaf Stuve reports a relationship with Genentech-Roche that includes: board membership. Olaf Stuve reports a relationship with Novartis that includes: board membership. Olaf Stuve reports a relationship with EMD Serono that includes: funding grants.

## Figures and Tables

**Supplementary Fig. 1:**
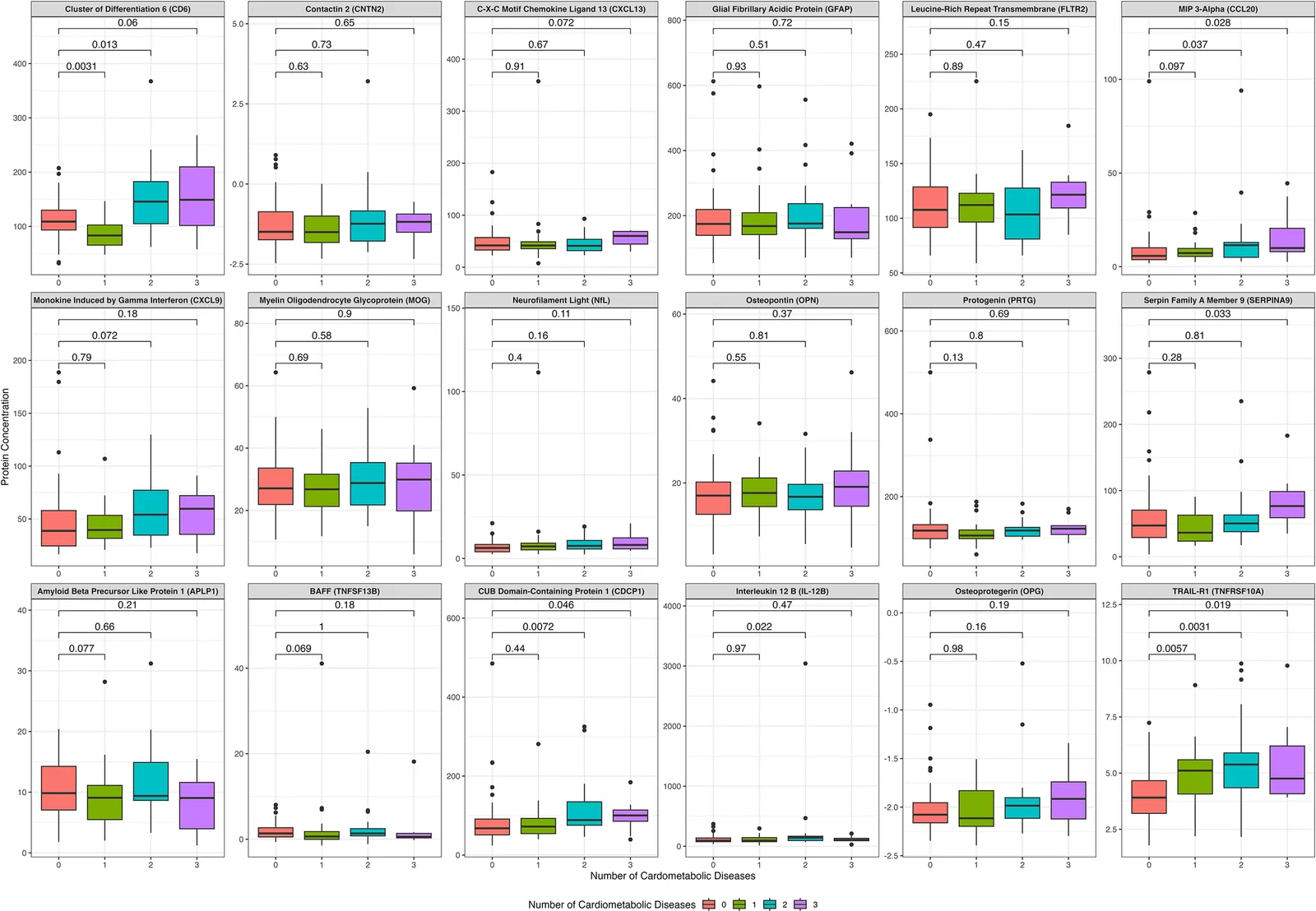
Boxplots showing the concentrations of the 18 individual proteins of interest in all untreated multiple sclerosis individuals. Individuals with no cardiometabolic disease (CMD) were compared to those with one, two, and three diseases to evaluate the impact of comorbidity burden on protein concentrations. P-values reflect pairwise comparisons adjusted for multiple testing.

**Supplementary Fig. 2:**
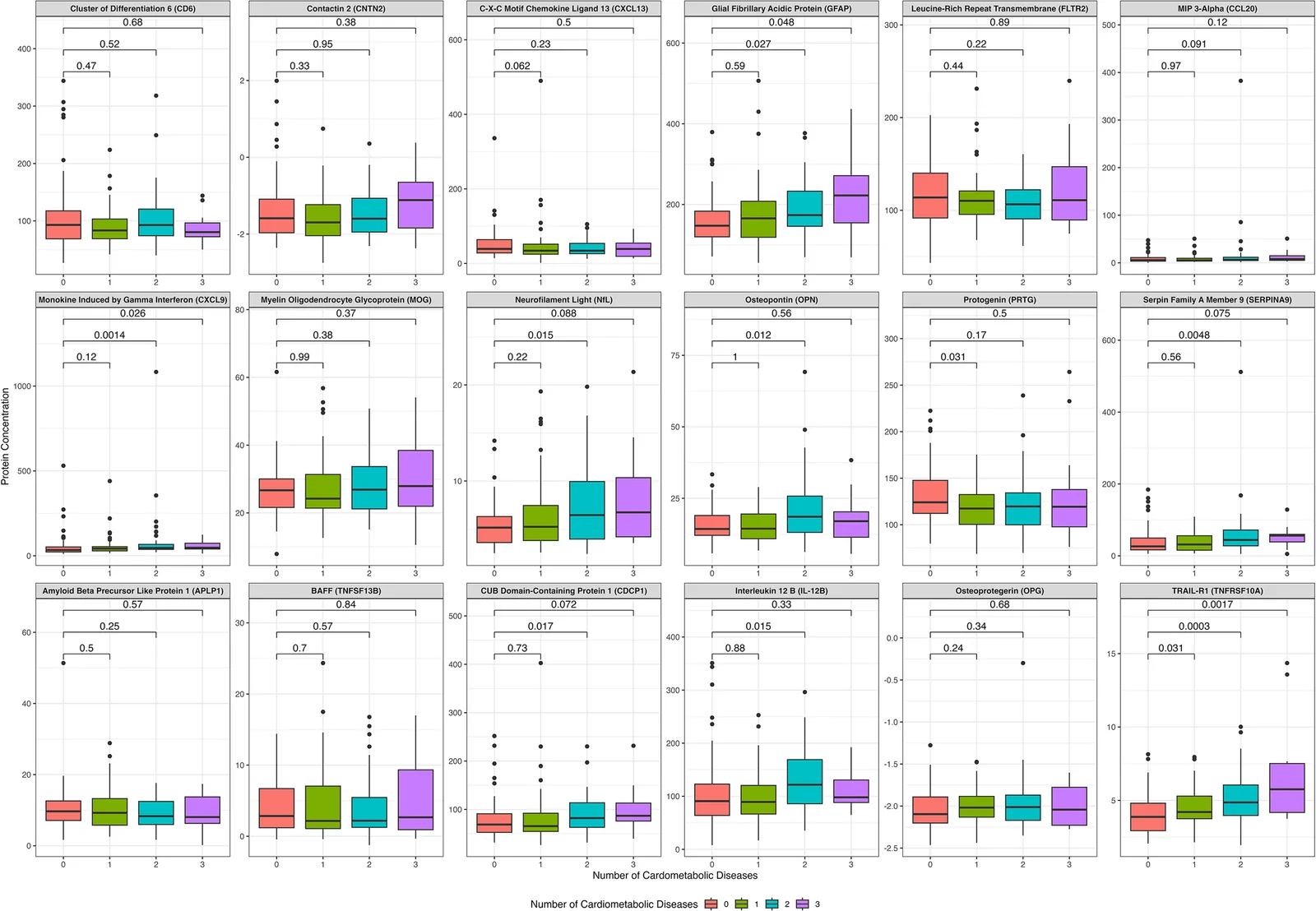
Boxplots illustrating the concentrations of the 18 individual proteins of interest in multiple sclerosis individuals treated with disease-modifying therapies. Individuals with no cardiometabolic disease (CMD) were compared to those with one, two, and three diseases to assess the cumulative impact of comorbidity burden on protein levels. P-values reflect pairwise comparisons adjusted for multiple testing.

## Appendix A Supplementary data

Supplementary Table 1 Completed STROBE reporting checklist.
